# Clinical and Neurocognitive Predictors of Functional Outcome in Depressed Patients with Partial Response to Treatment: One Year Follow-Up Study

**DOI:** 10.2147/NDT.S224754

**Published:** 2020-02-28

**Authors:** Sabrina Castellano, Carla Torrent, Maria Cristina Petralia, Justyna Godos, Rita Anna Cantarella, Andrea Ventimiglia, Simona De Vivo, Silvia Platania, Maria Guarnera, Concetta Pirrone, Filippo Drago, Eduard Vieta, Santo Di Nuovo, Dina Popovic, Filippo Caraci

**Affiliations:** 1 Department of Educational Sciences, University of Catania, Catania, Italy; 2 Barcelona Bipolar Disorders Program, Institute of Neurosciences, University of Barcelona, IDIBAPS, CIBERSAM, Barcelona, Catalonia, Spain; 3 IRCCS Centro Neurolesi Bonino Pulejo, Messina, Italy; 4 Oasi Research Institute-IRCCS, Troina 94018, Italy; 5 Department of Mental Health, ASP3 Catania, Catania, Italy; 6 Villa dei Gerani Clinic ASP3 Catania, Catania, Italy; 7 Faculty of Human and Social Sciences, University of Enna “KORE”, Enna, Italy; 8 Bipolar Disorders Program, Sheba Medical Center, Ramat Gan, Israel; 9 Department of Drug Sciences, University of Catania, Catania, Italy

**Keywords:** major depression, SSRI, SNRI, cognition, psychometric tools, antidepressant drugs

## Abstract

**Background:**

Cognitive dysfunction represents a distinct biological and clinical dimension in major depression disorders (MDD) and cognitive performance strongly affects psychosocial functioning in patients diagnosed with MDD.

**Objective:**

To assess which neurocognitive variables at baseline predict the functional outcome of MDD patients in a 1-year follow-up study as assessed by Functioning Assessment Short Test (FAST) and whether the improvement observed on affective and cognitive symptoms in our 12 week-prospective observational study after treatment with selective serotonin reuptake inhibitors (SSRIs) and selective noradrenalin reuptake inhibitors (SNRIs) can affect the following long-term psychosocial functional outcome at 1 year in the same MDD patients.

**Methods:**

We recruited a total of 31 patients (8 males; 23 females) with MDD who had previously completed a pharmacological treatment with SSRIs (n = 22) or SNRIs (n = 9) for 12 weeks, and then continued the same pharmacological treatment for 1 year. After an average 1-year follow-up, they were interviewed with the FAST to assess functional outcome. Multivariate analyses were applied to identify clinical and neurocognitive predictors of functional outcome.

**Results:**

Total Montreal Cognitive Assessment (MoCA), Digit Span forward (Span F) and backward (Span B), and 15 Rey words immediate recall (Rey I) scores significantly correlated with FAST. However, after performing regression models only Rey immediate recall score was useful to predict long-term functional outcome (Pearson correlation coefficient R= −0.68, p < 0.001) in four specific subdomains of FAST. When considering changes in affective and cognitive symptoms at the end of the 12 weeks of pharmacological treatment with SSRI or SNRIs (T1-T0) by multiple regression analysis, we found that Span *F*-test predicted scores in the FAST leisure domain, whereas, changes in Span F, Frontal Assessment Battery (FAB) and Rey I predicted psychosocial functioning in the specific “cognitive” subdomains of FAST.

**Conclusion:**

Our data suggest that long-term psychosocial functioning can be influenced by neurocognitive performance at baseline, with verbal memory playing a key role in overall functioning. Furthermore, improvement in verbal memory can predict functional outcome at one year in MDD patients with a recent history of partial response to antidepressants.

## Introduction

Major Depressive Disorder (MDD) is a severe mental illness that affects 5 to 20% of the general population.^1^,^2^ Epidemiological studies have shown that depression is estimated to affect 350 million people worldwide. The World Mental Health Survey conducted in 17 countries found that on average about 1 out of 20 people reported having an episode of depression in the previous year (World Health Organization, 2008).

The last version of the Diagnostic and Statistic Manual of Mental Disorder (DSM 5)^3^ describes MDD as a condition characterized by somatic and cognitive changes that significantly affect the individual’s capacity to function.^4^ Some authors have found a significant association between depression and disability.^5^,^6^

Cognitive dysfunction represents a distinct biological and clinical dimension in MDD. It is well known that cognitive performance strongly affects psychosocial functioning and, particularly, working ability in patients diagnosed with MDD^7^ as well as with bipolar disorder.^8^,^9^ A study conducted by Bonnìn et al^10^ on bipolar patients showed that depressive symptoms, combined with neurocognitive damage impairment related to executive functions and working memory, are predictors of long-term functional abilities. Other studies conducted in patients with bipolar disorder and aimed to evaluate the predictive value of cognitive impairment on long-term individual functioning have shown that both cognitive deficits and the duration of depressive episodes are associated with an impairment of functional abilities.^11^ Evans et al^12^ systematically reviewed the evidence on neurocognitive deficits and their relationships to psychosocial functioning in MDD, reporting that test performance in at least one cognitive domain was associated with a predicted functional outcome in all included studies.

Recently, Jha et al^13^ found that in depressed patients, although highly impaired at baseline, non-work-related activity significantly improves with treatment and also independently predicts long-term clinical outcomes.

A recent 12 week-prospective observational study in MDD patients with a recent history of partial response to antidepressants performed by our group indicated that Selective Serotonin Reuptake Inhibitors (SSRIs) and Serotonin and Noradrenaline Reuptake Inhibitors (SNRIs) can improve cognitive symptoms in MDD independently from their efficacy on affective symptoms,^14^ but it remains unclear whether or not the clinical improvement observed after a 12-week treatment can then affect functional abilities in a long-term perspective (1-year). In addition, cognitive side effects were reported in over 20% of patients suffering from depression or anxiety successfully treated with SSRIs over 6 months.^15^ Therefore, a long-term evaluation of antidepressants efficacy requires a long-term assessment of functional abilities.

The aim of the present follow-up study was to assess: i) which neurocognitive variables at baseline best predict the functional outcome of MDD patients in a 1-year follow-up study as assessed by Functioning Assessment Short Test (FAST); ii) whether the improvement observed on affective and cognitive symptoms in our 12 week-prospective observational study after treatment with SSRIs and SNRIs can affect the following long-term psychosocial functional outcome at 1 year in the same MDD patients.

## Methods

### Study Design

The study was approved by the ethical committees ASP3 Catania-Villa dei Gerani Clinic (July 24 2012). The study met the ethical administrative Italian legislation in force when the study administrative process started (03.06.2012) according to CM 6 02.09.2002, GU 214 12.09.2002 and D 29.03.2008 of the Italian Medicine Agency (Agenzia Italiana del Farmaco, AIFA) and GU 76 31.03.2008, Art 10 (Procedures for Observational Studies).

The designed study was a prospective, observational (non-interventional), cohort study. The study complied with the definition of “observational” (i.e. “non-interventional”) study provided in Article 2(c) of Directive 2001/20/EC, meaning that the investigator who carries out the study does not interfere with the physician’s decision regarding which drug is clinically pertinent to be prescribed to each individual patient. Therefore, prescription of SSRIs or SNRIs solely resulted from an independent clinical evaluation, according to the physician’s clinical judgment, and based on each patient’s clinical profile. Moreover, the decision to include a patient in the study, following his/her agreement, was taken independently of the clinical decision to prescribe SSRIs or SNRIs. Finally, the study did not affect the medical practice of participating physicians and did not trigger additional medical visits. In this respect, we carried out a 12 week-prospective observational study examining the effectiveness of SSRIs versus SNRIs in standard medical practice, in two cohorts of moderate-severe MDD patients, selected for their recent history of partial response to antidepressants (in the last 4 weeks). Partial response was evaluated at the beginning of the 12 week-prospective observational study and defined as a reduction in Hamilton Depression Rating Scale for Depression (HDRS) score ≥25% but <50%.

### Subjects

Patients were recruited at the Psychiatric Clinic “Villa dei Gerani” (Catania, Italy) and provided written informed consent. The study was conducted in accordance with the Declaration of Helsinki.

Thirty-one patients with MDD (8 males; 23 females), aged between 34 and 72 years, were selected for the present study from the larger sample of 49 partial responders MDD who previously completed our 12-week observational study conducted between 2014 and 2015.^14^

Patients were recruited with the following inclusion criteria: 1) MDD diagnosis according to DSM criteria (DSM-IV TR and DSM-5); 2) Age >30 and <75 years; 3) To have previously completed the observational study^14^ and the pharmacological treatment with SSRIs (n= 22) or SNRIs (n=9) for 12 weeks and, then, continued the same pharmacological treatment for 1 year (12 weeks of treatment during the observational study plus 9 months of follow-up).

Exclusion criteria were: 1) history of mental retardation or any clinical condition that could affect cognitive performance; 2) presence of manic or hypomanic episodes; 3) presence of other psychiatric disorders in comorbidity; 4) presence of other pharmacological treatments for psychiatric disorders (antipsychotics, mood stabilizers and benzodiazepines), and 5) presence of substance abuse/dependence.

The 22 MDD patients of the SSRI group completed the 12 weeks treatment (T2) and continued the pharmacological treatment up to 1 year with the following drugs: escitalopram (10 mg/day, n=11); paroxetine (20 mg/day, n=4), citalopram (20 mg/day, n=7). The remaining 9 MDD patients of the SNRI cohort completed the 12 weeks treatment with the following drugs: venlafaxine (225 mg/day, n=4), duloxetine (60 mg/day, n=5).

### Neuropsychological Assessment

During the observational study, patients underwent cognitive and neuropsychiatric assessments carried at baseline and at the end of the 12-weeks of pharmacological treatment. At baseline depressive symptoms were assessed by the Hamilton Depression Rating Scale (HDRS)^16^,^17^ and the Beck Depression Inventory (BDI–II).^18^ The patients were also assessed by a comprehensive neuropsychological battery consisting of: 1) Tools for the assessment of global cognitive function: Mini Mental State Examination (MMSE)^19^ and Montreal Cognitive Assessment (MoCA),^20^ and 2) Tools for the assessment of specific cognitive functions: Rey 15 Words Test^21^ and Verbal memory span (Digit-Span)^22^ was used to measure the short-term memory of participants^23^ and Frontal Assessment Battery to measure executive functions (FAB).^24^

The Functioning Assessment Short Test (FAST)^25^ was used as a primary outcome at study endpoint to identify predictors for specific functioning domains, such as: autonomy, occupational functioning, cognitive functioning, financial issues, interpersonal relationships and leisure time. The FAST was assessed after an average period of 1 year-follow-up after the beginning of the pharmacological treatment. For this study, we only analyzed the score of the overall functioning and the score of four specific FAST scale domains.

### Statistical Analyses

First, we preliminarily tested Spearman correlations to verify which clinical and neurocognitive variables at baseline were related to total FAST score at one year of follow-up. Rank correlation by means of Spearman test was selected because of the impossibility to assure the normality of distribution of all the variables necessary for using parametric tests. A threshold of P <0.1 was considered significant for selection of the variable for further investigation through linear regression analysis with both total FAST score and individual sub domains FAST scores as dependent variables. Subsequently, changes in affective and cognitive symptoms detected in MDD patients at the end of the observational study (12 weeks) were tested, after Bonferroni correction, through linear regression analyses adjusted for potential confounding factors [such as baseline clinical (e.g., depression severity, dosage) and demographic (e.g., age, gender) variables] to verify whether the clinical improvement observed at the end of the 12 weeks of pharmacological treatment affected psychosocial performance evaluated by FAST total score at one year of follow-up. All analyses were performed by software SYSTAT 12.0.

## Results

### Correlation Between Baseline Affective and Cognitive Symptoms and Psychosocial Functioning

The main sociodemographic and clinical characteristics of MDD patients are presented in Table 1.

**Table 1:** Sociodemographic and Clinical Characteristics of MDD Patients

	Patients (n = 31)
Age; mean (SD)	54.71 (12.04)
MMSE baseline, mean (SD)	25.31 (4.30)
HDRS baseline mean (SD)	22.55 (7.38)
BDI baseline, mean (SD)	33.29 (11.91)
Scholar, years	8.77 (3.73)
Sex, n (%)	
Male	8 (25.8)
Female	23 (74.2)
Antidepressant therapy, n (%)	
SSRI	22 (71)
SNRI	9 (29)

We first analyzed the correlation between cognitive and affective symptoms at baseline and FAST total score one year after the beginning of the pharmacological treatment in the 22 MDD patients treated with SSRIs and in the 9 MDD patients treated with SNRIs. In particular, we considered MMSE, MoCA scores for global cognitive function, FAB scores for executive function, Span forward, Rey’s 15 Words Test and delayed Rey test scores for verbal memory, Span backward scores for working memory, whereas HDRS and BDI–II were used for baseline affective symptoms. The FAST total score was negatively significantly correlated with FAB, MoCA, SPAN at baseline in the unadjusted model, but only the MoCA (r_s_= −0.48; p=0.06), SPAN forward (r_s_= −0.46; p=0.08), and Rey immediate recall scores (r_s_= −0.66; p<0.001) were considered for further analyses. No other correlations were found between FAST total score and baseline MMSE, Span Backward, delayed Rey test scores for verbal memory and psychosocial functioning at 1 year follow up.

When considering baseline affective symptoms, no correlation was found with FAST total scores. In particular, a trend of positive correlation was detected with BDI baseline scores, whereas a negative correlation was found between baseline HDRS scores and FAST total scores at 1 year (Table 2). Interestingly, we found a high positive correlation between the BDI baseline score and the FAST cognitive functioning domain at 1 year of follow-up (r=0.33; p<0.05). Besides, a significant negative correlation was found between the HDRS baseline test score and the financial capacities (r=0.36; p<0.05). An inverse, although not significant, correlation was observed between total FAST and HDRS scores.

**Table 2:** Correlations Between Baseline Cognitive Variables and Total FAST Score

	FAST	p	p*
MMSE_T0	−0.42	**0.02**	0.18
MOCA_T0	−0.48	**0.01**	**0.06**
FAB_T0	−0.39	**0.03**	0.26
SPAN_F_T0	−0.46	**0.01**	**0.08**
SPAN_B_T0	−0.43	**0.02**	0.14
REY_I_T0	−0.66	**0.00**	**0.00**
REY_D_C_T0	−0.23	0.22	1.00

**Notes:** *After Bonferroni correction. Bold values indicate statistical significance.

### Regression Models

**Figure 1:**
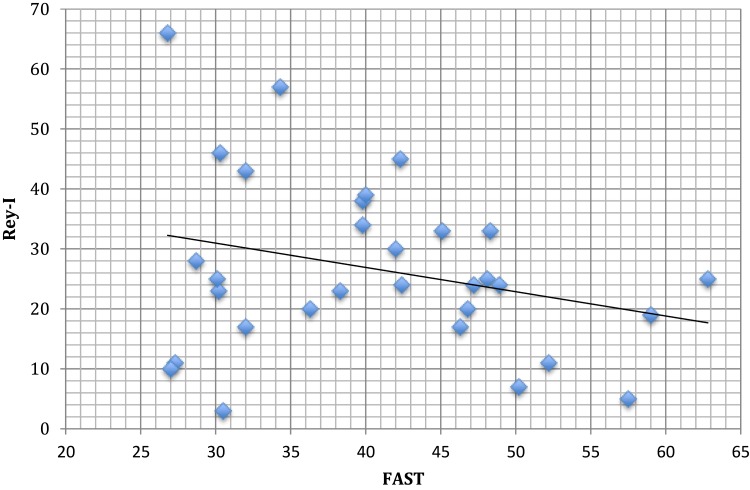
Plot of the relationship between Rey immediate recall scores (Rey-I) and FAST.

On the basis of the preliminary correlation analysis, using the variables significantly correlated with FAST (i.e. total MoCA, SPAN forward, and Rey immediate recall scores), we then performed regression models to assess which cognitive variables at baseline better predict functional outcome at one year follow-up. Only Rey immediate recall score was useful to predict long-term functional outcome (beta = −0.68, p<0.001). More in detail, Rey immediate recall score predicts psychosocial functioning in 4 specific subdomains of FAST, i.e. the autonomy domain, the FAST financial domain, the FAST interpersonal domain and the leisure domain (Table 3, Figure 1).

**Table 3:** Multiple Regression Analysis Between Cognitive Tests and FAST at 1 Year of Follow-Up

	Autonomy		Financial		Interpersonal		Leisure	
	R^2^ 0.47		R^2^ 0.41		R^2^ 0.27		R^2^ 0.29	
	Std. Coeff.	p-value	Std. Coeff.	p-value	Std. Coeff.	p-value	Std. Coeff.	p-value
MOCA	−.12	.65	.36	.19	.18	.55	.50	.10
SPAN_F	−.26	.23	−.41	.07	−.02	.93	−.21	.37
REY_I	−.58	.002	−.57	.005	−.41	.05	−.62	.01

Subsequently, we examined whether changes in affective and cognitive symptoms at the end of the 12 weeks of pharmacological treatment with SSRI or SNRIs (T1-T0) were associated with overall psychosocial functioning at 1 year of follow up as assessed by the total FAST score. We found that the change detected in the Span forward test correlated with FAST total score (r_s_= 0.47) and the differences detected between T1 and T0 with the Rey immediate recall *t*-test (r_s_= 0.47), although after Bonferroni correction both were not significant. We then performed linear regression analyses to establish which of the three cognitive tests (Span F, FAB, ReyI) better predicted FAST total score. We found that only changes in scores from Span forward test were able to predict psychosocial functioning (Table 4). In particular, changes in scores from Span forward test after 12 weeks predicted scores in the FAST leisure domain, whereas changes in all of the three cognitive tests (Span F, FAB, Rey I) predicted psychosocial functioning in the specific “cognitive” subdomains of FAST.

**Table 4:** Multiple Regression Analysis Between Improvement in Cognitive Tests and FAST at 1 Year of Follow-Up (T1-T0)

	Autonomy		Interpersonal		Leisure		Cognitive		Total	
	R^2^ 0.18		R^2^ 0.30		R^2^ 0.24		R^2^ 0.47		R^2^* .*0.37	
	Std. Coefficient	p-value	Std. Coefficient	p-value	Std. Coefficient	p-value	Std. Coefficient	p-value	Std. Coefficient	p-value
FAB (T1-T0)	0.98	0.58	0.27	0.10	0.19	0.28	0.41	0.01	0.29	0.06
SPAN_F (T1-T0)	0.35	0.06	0.39	0.03	0.46	0.01	0.41	0.01	0.40	0.02
REY_I (T1-T0)	0.01	0.94	0.27	0.12	−0.04	0.84	0.31	0.02	0.28	0.09

Finally, we detected, despite not significant, a trend of correlation *(Rs* 0.33; p=0.07) between changes in HDRS scores at 12 weeks of treatment and total FAST scores, whereas no correlation was found between changes in BDI scores and psychosocial functioning.

## Discussion

Results of the present study indicate that neurocognitive performance at baseline, particularly verbal memory, affects long-term psychosocial functioning.

Besides, in MDD patients with a recent history of partial response to antidepressants, functional outcome at one year can be predicted by the improvement in verbal memory.

Cognitive deficits are considered as key symptoms of clinical depression associated with reduced psychosocial functioning in MDD patients^26^ and in particular with deficits in working ability.^7^ Two important open questions still remain: a) whether cognitive factors at baseline (before initiating a pharmacological treatment) can predict the functional outcome of MDD patients in 1-year follow-up study, and b) whether clinical improvement in cognitive symptoms after an adequate treatment with second-generation antidepressants drugs can influence long-term psychosocial functioning in MDD patients.

In the present study, we found that only specific cognitive factors, such as free delayed recall (verbal memory), are significant predictors of long-term functional outcome in MDD patients as assessed at 1 year of follow up with a specific functioning scale (FAST). FAST is a psychometric tool developed to assess psychosocial functioning in patients with bipolar disorder and validated in clinical samples.^27^,^28^ It is known that cognitive reserve is correlated with psychosocial functioning and, thus, with FAST score in bipolar patients.^10^ Other studies demonstrated a cross-sectional link between verbal memory and poor overall functioning in bipolar patients.^29^–^31^

The present study is the first long-term study demonstrating the predictive value of neurocognitive performance (Rey immediate recall score) on long-term functional outcome in unipolar-depressed patients. In particular, Rey immediate recall score predicted psychosocial functioning in different specific subdomains of FAST such as autonomy, financial, interpersonal domain and leisure domains (Table 3). Although a trend of correlation was found with MoCA and SPAN forward scores, statistical significance was not reached probably due to the small sample size.

Cognitive domains have been shown to have considerable impact on vocational functioning including deficits in memory, attention, learning and executive function.^32^ However, in the present study executive dysfunction did not predict psychosocial functioning. Recent studies demonstrated that deficits in executive functioning in MDD are associated with psychosocial outcomes as assessed by FAST.^33^ Future studies with larger sample size and longer follow-up are needed to better understand the predictive value of this cognitive factor in MDD.

Interestingly, no correlation was found between psychometric tests assessing affective symptoms at baseline (HDRS, BDI) and functional outcome at one year. Furthermore, the scores from BDI showed a trend of positive correlation with FAST total scores as opposite to HDRS scores, suggesting that the adopted instrument (self-report versus rating scale) can strongly affect data analysis and results. These results also suggest that the severity of cognitive symptoms probably exerts a more relevant role compared to affective symptoms in determining long-term functional abilities.

Another finding of our study is that changes in cognitive symptoms (i.e. verbal memory) continue to affect psychosocial functioning after one year of treatment. Interestingly, changes in three different cognitive tests (Span F, FAB, Rey I) were able to predict psychosocial functioning in the specific “cognitive” subdomains of FAST, suggesting that this tool is able to detect deficits in functional abilities strictly related to specific cognitive deficits in MDD.

The present study has some limitations intrinsically to the observational design (i.e. absence of randomization and of control group, no treatment-intervention, groups not matched for number, sex and treatment) and the duration of the study. Other limitations are small sample size and the heterogeneity of the pharmacological treatments (two pharmacological classes of antidepressants and four different molecules not equally distributed in the sample). Moreover, the study did not include during the observational period any treatment with vortioxetine, which is one of the few antidepressants with positive primary effects on cognitive performance,^34^ because the drug was not marketed at that time. Thus, further studies are needed in order to validate these findings.

Taken together, our data suggest that long-term psychosocial functioning can be influenced by neurocognitive performance at baseline, with verbal memory playing a key role in overall functioning. Thus, clinicians, preliminarily to pharmacological treatment, should consider to assess cognitive functions at base. Furthermore, improvement in verbal memory can predict functional outcome at one year in MDD patients with a recent history of partial response to antidepressants.
